# Chatting your way to quitting: A longitudinal exploration of smokers' interaction with a cessation chatbot

**DOI:** 10.1016/j.invent.2025.100806

**Published:** 2025-02-04

**Authors:** Linwei He, Erkan Basar, Reinout W. Wiers, Marjolijn L. Antheunis, Emiel Krahmer

**Affiliations:** a Department of Communication and Cognition, Tilburg School of Humanities and Digital Sciences, Tilburg University, Tilburg, the Netherlands; b Behavioral Science Institute, Radboud University Nijmegen, Nijmegen, the Netherlands; c Addiction Development and Psychopathology (ADAPT)-lab, Department of Psychology, University of Amsterdam, the Netherlands; d Centre for Urban Mental Health, University of Amsterdam, the Netherlands

**Keywords:** Conversational agents, Chatbots, Smoking cessation, Engagement, Longitudinal evaluation, User experience

## Abstract

**Background:**

Cigarette smoking poses a major public health risk, requiring scalable and accessible interventions. Chatbots offer a promising solution, given their potential in providing personalized, long-term interactions. Despite their promise, limited research has examined their efficacy and the intertwined relationship between user experience and effectiveness over an extended period of time.

**Methods:**

In this prospective, single-arm study, we developed and evaluated *Roby*, a 5-session chatbot intervention incorporating motivational interviewing and cognitive behavioral therapy to help smokers quit. *Roby* engaged Dutch adult smokers (*N* = 102) in conversations covering topics such as setting a quit date, managing withdrawal and cravings, and relapse prevention. The primary outcome was the continuous abstinence rate at the end of the intervention, and secondary outcomes included 7-day point prevalence abstinence, self-efficacy, and cravings. User engagement, therapeutic alliance, and interaction satisfaction were measured weekly, and the trajectory was analyzed using Linear Mixed Models.

**Results:**

Following an intention-to-treat principle, 18.6 % of participants achieved continuous abstinence, and 37.3 % achieved 7-day point prevalence abstinence. Self-efficacy significantly improved over the intervention, and cravings decreased over time. A slight decreasing trend was observed in engagement and satisfaction, likely due to a novelty effect. However, the decrease did not affect the intervention's outcomes.

**Conclusion:**

This study demonstrates the feasibility and initial usefulness of *Roby*, highlighting the potential for chatbots in long-term cessation support. Future research should further validate these findings with randomized controlled trials. Additional efforts should focus on monitoring and maintaining user experience in the long term to enhance effectiveness.

## Highlights

• Developed Roby, a longitudinal chatbot intervention for smoking cessation using MI and CBT techniques
• Session-by-session analysis reveals patterns in cessation outcomes and user engagement.
• Achieved 18.6 % continuous abstinence, with significant over time increase in self-efficacy and reduction in cravings
• Slight drop in user engagement, likely due to novelty effect, did not impact intervention outcomes.

## 1 Introduction

Smoking cigarettes is a leading preventable cause of illness and premature death worldwide (WHO, 2023). In the Netherlands, where this study took place, 19 % of the adult population smoke cigarettes, and smoking-induced diseases account for over 19,000 deaths annually (Trimbos Instituut, 2023). According to a recent nationwide survey, 34.4 % of smokers made a serious attempt to quit in 2023 (Trimbos Instituut, 2023). To support smokers to quit, various cessation methods are available, ranging from pharmacological support such as nicotine replacement therapy to behavioral support such as telephone quit lines and face-to-face counseling (Stead et al., 2016). While effective, these methods have limited reach due to staff shortages, resource constraints, and smokers' reluctance to seek help, often driven by stigma or the effort required for repeated visits (Huddlestone et al., 2022; Patel et al., 2018). As a result, unassisted quit attempts typically yield lower success rates (Stead et al., 2016).

To address these challenges, innovative digital tools such as chatbots have emerged as a popular approach for providing cessation support. Chatbots are computer programs that simulate conversations with human users through natural language (Adamopoulou and Moussiades, 2020). Research suggests that chatbots can offer personalized and more engaging experiences than traditional methods, enhancing long-term support (Shaffer et al., 2020). These opportunities have led to an increased interest in exploring the use of chatbots in healthcare settings. Studies have shown initial effectiveness in various areas, such as promoting healthy lifestyles, improving medication adherence, and supporting mental health (Aggarwal et al., 2023). The use of chatbots for smoking cessation is still in its early stage but holds great potential. A systematic review, although based on only 6 randomized controlled trials (RCTs), found an overall positive effect on abstinence and high user acceptability (He et al., 2023). However, the authors highlighted several methodological limitations, including the lack of reporting on theoretical foundation and technical infrastructure, as well as variations in the collection and measures of user experience. A more recent review reported that chatbots are often integrated into other intervention content (e.g., social media, mobile applications) (Bendotti et al., 2023), making it difficult to have an isolated evaluation of the chatbot. As a result, while scientific interest is growing, the true potential of chatbots in engaging users for long-term cessation support remains inconclusive, calling for more rigorous development and evaluation to ascertain the feasibility and effectiveness of chatbot interventions.

Furthermore, little research has examined the intertwined relationship between user experience and effectiveness, while their interplay holds an essential role in long-term usage. Engagement with digital interventions has proven to be a significant predictor of sustained usage and therefore positive behavioral change (Perski et al., 2017). In the field of human-computer interaction, research highlights the dynamic and experiential nature of user engagement in the long run, such that a user's subjective experience of engagement can vary over multiple interaction sessions or even within one session (Doherty and Doherty, 2019). However, many studies in chatbot interventions simply measure user engagement as the amount of usage (e.g., number of interaction sessions), overlooking the subjective experience of the users over time (He et al., 2023). Building a therapeutic alliance is another challenge for chatbot interventions. A positive alliance is a robust predictor of treatment outcomes and is especially important for long-term behavior changes such as smoking cessation which requires sustained effort (Connors et al., 1997). However, whether a therapeutic alliance can be formed and sustained in the digital context is unclear. Some studies showed that users could potentially build a bond with a mental health chatbot (Tong et al., 2022), but little research has explored this concept in the smoking cessation context. In our earlier studies (He et al., 2022, He et al., 2024a), we observed that a chatbot can foster a sense of engagement and empathy in the short term (with two interaction sessions). However, research in the broader fields of human-computer interaction suggests that such effects may decline over time as the novelty of the interaction wears off (Croes and Antheunis, 2021; De Haas et al., 2022). Despite this, there is limited research of how this long-term dynamic unfolds in the context of health interventions. Given the novelty of chatbot interventions for smoking cessation, it is essential to have a better understanding of the trajectory of user experience as part of the intervention outcomes. This understanding will help in developing more effective and engaging chatbot-based support for smoking cessation.

To address these considerations, we designed a 5-week chatbot intervention for smoking cessation. This study has two parallel objectives. The primary aim is to assess the feasibility of and user experience with the chatbot intervention. The second aim is to examine the usefulness for smoking cessation, particularly in self-reported abstinence, craving, and self-efficacy. We give special attention to the trajectory of user experience and the quitting process, aiming to identify usage patterns and their relationship with cessation outcomes.

## 2 Methods

### 2.1 Study design and participant recruitment

This was a single-arm intervention study conducted to examine the feasibility and usefulness of Roby, a chatbot designed for smoking cessation. The intervention consisted of 1 pre-quit session and 4 weekly follow-up sessions. We examined the levels of engagement, therapeutic alliance, and general satisfaction with the chatbot interactions, analyzed the changes over time, and described self-reported smoking cessation as the intervention outcome.

We recruited adult smokers in the Netherlands from April 2024 to July 2024. Recruitment occurred through (1) participant pools from the Tilburg School of Humanities and Digital Sciences and Tilburg School of Social and Behavioral Sciences; (2) Prolific, a research participant website; and (3) social media advertisement on Facebook and Instagram. To be eligible, participants had to: (1) be aged 18 or older; (2) be an active smoker (i.e., had smoked at least one cigarette in the week before participation); (3) want to quit smoking in the next 2 weeks; and (4) not be participating in any other cessation interventions. These eligibility criteria were based on earlier studies and were designed to enable recruitment of a broad range of smokers, aligning with the exploratory nature of this intervention (Hennrikus et al., 2005; Naughton et al., 2014; Strecher et al., 2008). Participants received either course credits or a gift voucher worth €10 as a token of appreciation. This study was approved by the Research Ethics and Data Management Committee of the Tilburg School of Humanities and Digital Sciences (identification code REDC2021.18d) and was conducted in compliance with the ethical and data management regulations of the school. All participants provided informed consent via the Qualtrics (Qualtrics International Inc) web form.

### 2.2 Chatbot content and development

#### 2.2.1 Content

**Fig. 1:**
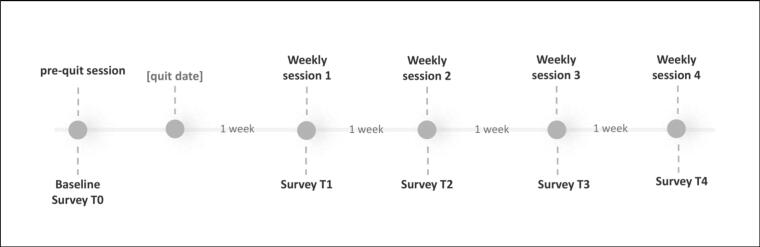
The intervention timeline.

Roby was designed by a multidisciplinary team from the fields of smoking cessation, communication science, and computational linguistics. This project has been under development for several years, with continuous iterations and improvements of the chatbot prototypes. Participants had 1 pre-quit session and 4 weekly sessions with Roby, as depicted in Fig. 1. The pre-quit session was based on a previous chatbot prototype, where the counseling approach was based on Motivational Interviewing (MI) (Miller and Rollnick, 2012). In this session, the chatbot encouraged smokers to reflect on their intrinsic motivations, discussed quitting experiences and personal strengths to increase self-efficacy, and guided them to make a quit plan and set a quit date. The prototype was previously tested, and we incorporated user feedback to improve the content. A more detailed description can be found elsewhere (He et al., 2024a). The 4 weekly sessions following the quit date incorporated elements of cognitive behavioral therapy (CBT). We used a manual-guided protocol (Duhig et al., 2003; Kong et al., 2015) adapted for the adult population to draft the conversation content.^1^ Each session focused on a specific theme, as described below:

• **Week 1: Coping with withdrawal symptoms**. Participants were guided to identify and manage withdrawal symptoms through techniques such as alternative activities for distraction and the decision-delay technique, where cravings were framed as temporary “waves” to be endured for 15 min.
• **Week 2: Problem-solving skills.** The chatbot used role-playing exercises to help participants identify high-risk situations and practice problem-solving skills to effectively avoid smoking.
• **Week 3: Managing negative emotions.** Participants were guided to identify emotional triggers, challenge unhelpful thoughts, and create a list of enjoyable activities to manage stress without smoking.
• **Week 4: Sustain a smoke-free lifestyle.** The chatbot revisited all the topics and skills discussed in previous weeks. This session concluded with an imagery exercise where participants envisioned a smoke-free future and reflected on the long-term benefits of quitting to strengthen their motivation.

The design and content of the weekly sessions aligned with the relapse prevention framework, progressively introducing interconnected skills that support smoking abstinence (Hajek et al., 2013). Each session started with a review of the previous week, followed by the chatbot eliciting positive experiences and discussing obstacles encountered, after which the chatbot introduced the topic of the week. The dialogue content protocol was registered at the Open Science Framework (He, 2024). Each session lasted 10–15 min. The content development followed an iterative process involving regular collaborative consultation with the team. The chatbot was offered in both English and Dutch.

The chatbot content adhered to the Dutch national guideline for smoking cessation in primary care (Verbiest et al., 2017) except for offering pharmacological and human behavioral support. For any discussions related to medication, the chatbot directed participants to a public health website designed to support smoking cessation (Trimbos Instituut, 2024) for further information.

Participants were provided with access to the chatbot throughout the study period. If they reached out to the chatbot between the weekly sessions, the chatbot provided information on frequently discussed topics (e.g., managing cravings) by referring to the public health smoking cessation website (Trimbos Instituut, 2024). After all the scheduled sessions, participants still had access to the chatbot, which provided the option to review and discuss the weekly topics.

#### 2.2.2 Technicality

**Fig. 2:**
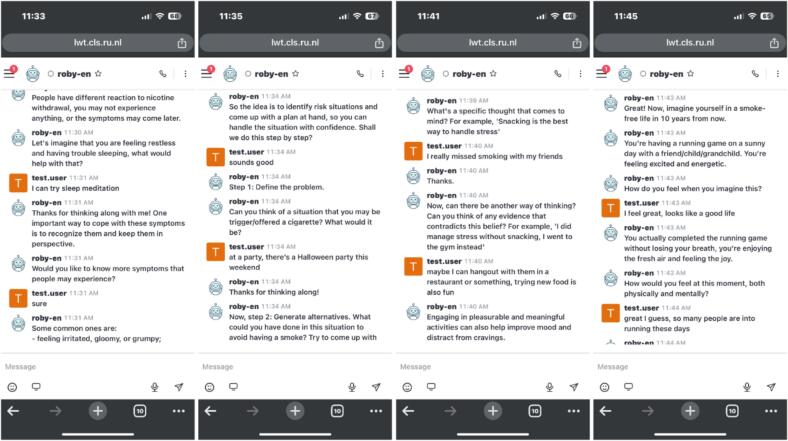
Screenshots of the chatbot interface from weekly session 1 to 4.

The dialogue content was programmed using an open-source framework HyLECA (Basar et al., 2023), developed to build chatbots capable of holding long-term engaging conversations with users. Roby was equipped with a hybrid architecture, combining rule-based methods for controlled dialogue flows and retrieval-based approaches to enhance the utterance variability and flexibility. Roby was primarily pre-scripted by the author teams to ensure that the content followed clinical guidelines and to avoid potentially inaccurate and even harmful information that might be generated by uncontrolled large language models (Bender et al., 2021). To increase variability, we wrote multiple alternative utterances where applicable, and Roby selected the best-fitting utterance at each turn based on the topic, user data, and conversation history. A detailed description of the technical infrastructure is provided elsewhere (Basar et al., 2023). Roby operated on the Rocket.Chat web interface, which can be accessed on mobile phones, tablets, and computers. Fig. 2 shows an illustration of the interface.

### 2.3 Measures

#### 2.3.1 Baseline measures

Demographic variables (age, gender, and education) were measured using single items at T0. Smoking behavior measures included years of smoking, whether the participant smokes daily, number of quit attempts made in lifetime, and daily cigarette consumption. Level of nicotine dependence was measured using the Fagerström Test for Cigarette Dependence (Fagerström, 2012). Baseline motivation to quit was assessed using the Contemplation Ladder (Biener and Abrams, 1991), where participants indicated their motivational status ranging from 0 (no thoughts on quitting) to 10 (taking action to quit).

#### 2.3.2 User experience measures

User experience was measured weekly after each interaction session (T0–T4) using the following variables. All scale variables were measured on 5-point scales.

*Engagement*. Assessed using the short form of the User Engagement Scale (O'Brien et al., 2018), excluding the aesthetic appeal subscale as this study focused on the communication process rather than interface design. An example item is: “I was absorbed in this experience.” An additional question asked participants about their endorsement for future use.

*Therapeutic alliance*. Measured using the Working Alliance Inventory - Short Revised (Hatcher and Gillaspy, 2006). An example item is: “We agree on what is important for me to work on.”

*Interaction satisfaction*. Measured using 5 items following studies on telephone smoking cessation counseling (Rogers et al., 2016). An example item is: “how was your counsellor in terms of being a good listener?”

#### 2.3.3 Intervention outcome measures

The primary outcome measure was self-reported continuous abstinence (not smoking since the quit date), measured at the end of the intervention (T4). The secondary outcome measure was self-reported 7-day point prevalence abstinence (7-day PPA; not smoking for 7 days before the time of measurement).

We also measured the following variables weekly (T0–T4):

*Self-efficacy*. Assessed using the Smoking Self-Efficacy Questionnaire (Etter et al., 2000). Participants indicated on a 5-point scale their confidence in refraining from smoking in various difficult situations. An example question is: “how sure are you that you could refrain from smoking when you feel nervous.”

*Craving*. Assessed with a single question “How much of the time have you felt the urge to smoke today?”, with answer ranging from “none at all” to “all the time” (West and Ussher, 2010).

### 2.4 Statistical analyses

Data analysis was conducted using R (version 4.2.1; R Foundation for Statistical Computing). The primary analysis on cessation followed an intention-to-treat approach, incorporating all available data and categorizing participants lost to follow-up as smokers. Abstinence rates were reported descriptively. Weekly measurements of self-efficacy and craving were analyzed using Linear Mixed Models (LMMs) with time (i.e., session) as a fixed effect and participants as a random effect. Maximum-likelihood estimation was used to account for missing data, and *p*-values were obtained using Satterthwaite's approximation method (Kuznetsova et al., 2014). To gain a more comprehensive understanding of the trajectory over time, pairwise comparisons were performed using Tukey contrasts to compare between time points.

The progression of user experience (engagement, therapeutic alliance, and interaction satisfaction) was similarly examined using LMMs for each variable. Additionally, to explore the potential relationship between user experience and abstinence outcome, in each model we included time, continuous abstinence status, and their interaction as fixed effects, with participants treated as a random effect. This approach investigated the overall trend in user experience and whether the changes are associated with cessation outcomes, thereby providing insights into the experience patterns that may contribute to successful quitting.

Additionally, two exploratory logistic regression analyses were conducted. The first analysis investigated factors associated with dropout, and the second focused on predictors of self-reported abstinence. Explanatory variables included demographic characteristics and baseline smoking behaviors.

We replicated these analyses among participants with complete data, and no significant differences were found between these results and those from the full sample analysis.

## 3 Results

### 3.1 Sample characteristics

**Fig. 3:**
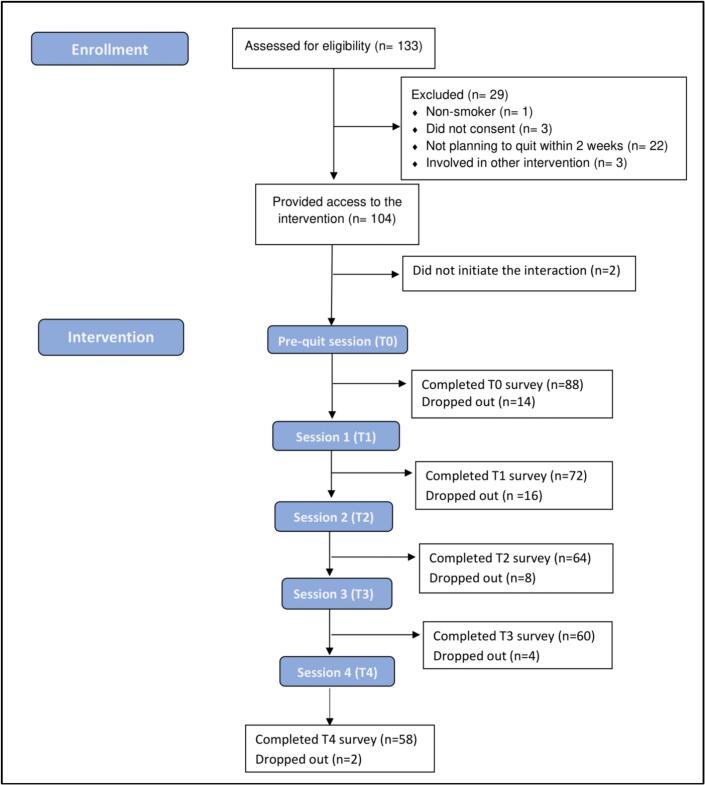
Modified CONSORT (Consolidated Standards of Reporting Trials) flow diagram.

Fig. 3 presents the flow of participants from enrollment to post-intervention. Of the 133 respondents assessed for eligibility, 1 (0.8 %) was nonsmokers at baseline, 3 (2.3 %) declined to participate, 22 (16.5 %) were not motivated to set a quit date within 2 weeks, and 3 (2.3 %) were using other forms of cessation intervention. As a result, 104 respondents were provided access to the chatbot, and 102 of them initiated interaction and completed baseline measures. These 102 participants had a mean age of 24.7 years; 54 (53 %) were female; and 64 (62.7 %) had a medium level of education. Regarding baseline smoking behaviors, 46 (45.1 %) participants smoked daily. The majority (80, 78.4 %) were light smokers (smokes 10 cigarettes or less per day), and 81 (79.4 %) reported low levels of nicotine dependence. See Table 1 for demographic characteristics and baseline smoking behavior information.

**Table 1:** Baseline characteristics of participants (*n* = 102).

Profile characteristics	Statistics
**Age, mean (SD)**	24.7 (9.95)
**Gender**	
Female	54 (53.0 %)
Male	46 (45.0 %)
Prefer not to say	1 (1.0 %)
Non-binary	1 (1.0 %)
**Educational level, n (%)**	
Low (Primary and secondary education)	1 (1.0 %)
Medium (High school and vocational education)	64 (62.7 %)
High (University education)	37 (36.3 %)
**Smoking pattern, n (%)**	
Daily	46 (45.1 %)
Nondaily	54 (52.9 %)
**Years of smoking, mean (SD)**	6.22 (7.02)
**Daily cigarette consumption, mean (SD)**	7.05 (5.22)
**Fagerström test for cigarette dependence, n (%)**	
Low dependence	81 (79.4 %)
Moderate dependence	19 (18.6 %)
High dependence	0
**Baseline motivation to quit, mean (SD)**	6.38 (2.24)
**Number of lifelong quit attempts, mean (SD)**	3.66 (3.88)

### 3.2 Intervention outcomes

Self-report continuous abstinence since quit date and 7-day PPA were collected postintervention at T4. Following the intention-to-treat principle, participants lost to follow-up were considered smokers. By the end of the intervention, 18.6 % of the participants (19/102) reported complete abstinence since the quit date, and 37.3 % (38/102) reported not smoking in the past 7 days. Of the 58 participants who completed the intervention, 32.8 % (19/55) achieved continuous abstinence and 65.5 % (38/58) achieved 7-day PPA.

We conducted an exploratory logistic regression analysis to identify factors associated with achieving abstinence. However, none of the demographic variables or baseline smoking behavior appeared to be significant predictors of abstinence. Detailed results can be found in the online supplementary file.

**Fig. 4:**
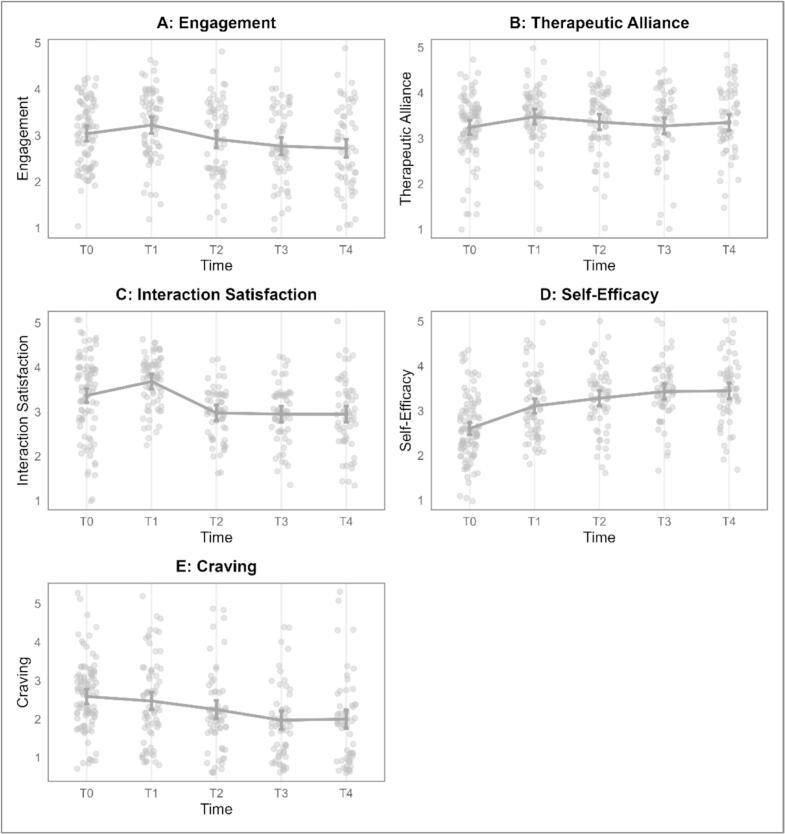
Trajectories of outcome variables over time, with individual data points plotted. Panels A to E represent Engagement, Therapeutic Alliance, Interaction Satisfaction, Self-Efficacy, and Craving, respectively. Error bars indicate 95 % confidence intervals.

Regarding self-efficacy and experienced cravings, results of a LMM showed a significant increase in self-efficacy from baseline to post-intervention (*b* = 0.21, *p* < 0.001). Post-hoc Tukey contrasts revealed that the most notable improvement occurred between the pre-quit session (T0) and the first weekly session (T1), with a 0.51-point rise (*p-adj* < 0.001). This increase continued through to T4, resulting in a total 0.84-point increase (*p-adj* < 0.001). Fig. 4 illustrates the estimated marginal means at each time point alongside individual data points. Detailed estimated marginal means are provided in Table 2. A reversed trajectory was observed for cravings, showing an overall decrease from baseline to post-intervention (*b* = −0.17, *p* < 0.001). The first significant drop was from T0 to T3, marked by a 0.62-point decrease (*p-adj* < 0.001), which was maintained at T4.

**Table 2:** Summary of outcome variables at each time point*.*

Outcome measure	T0 Mean (SD)	T1 Mean (SD)	T2 Mean (SD)	T3 Mean (SD)	T4 Mean (SD)
Self-efficacy	2.60 (0.66)	3.11 (0.67)	3.28 (0.68)	3.43 (0.68)	3.44 (0.68)
Craving	2.59 (0.92)	2.47 (0.96)	2.25 (0.95)	1.97 (0.94)	2.00 (0.94)
Engagement	3.12 (0.96)	3.22 (0.89)	2.94 (0.85)	2.77 (0.83)	2.77 (0.82)
Therapeutic Alliance	3.26 (0.91)	3.49 (0.83)	3.42 (0.80)	3.28 (0.78)	3.39 (0.77)
Interaction satisfaction	3.43 (0.90)	3.69 (0.83)	2.99 (0.80)	2.95 (0.79)	2.97 (0.78)

### 3.3 User experience and its relationship with abstinence

*Engagement*. Results from the LMM showed a slightly declining trend over the course of the intervention (*b* = −0.12, *p* = 0.002). Engagement levels remained consistent across the first three sessions, with the first notable, albeit modest, dip observed at T3 (0.35-point reduction from T0, *p-adj* = 0.008). The decrease remained robust at post-intervention, with a total 0.35-point decrease from T0 (*p-adj* = 0.010). The analysis further revealed that this general decline in engagement did not differ among participants who achieved abstinence and those who did not. The interaction between time and abstinence status was not significant (*b* = 0.03, *p* = 0.580), suggesting that the trajectory of engagement was similar across both groups. Therefore, engagement with the chatbot interactions might not be an influential factor in cessation.

*Therapeutic alliance*. No significant overtime change was found for therapeutic alliance (*b* = 0.02, *p* = 0.558). Participants reported a moderate level of alliance at T0 (*M* = 3.26, *SD* = 0.91), and this level remained stable through to T4 (*M* = 3.39, *SD* = 0.77). This stable level was similar among smokers and those who achieved abstinent (*b* = −0.09, *p* = 0.629), suggesting that the experience of therapeutic alliance did not significantly impact whether a participant successfully quit smoking or not.

*Interaction Satisfaction*. A small yet significant decrease was observed in participants' satisfaction with the interactions (*b* = −0.18, *p* < 0.001). Tukey contrasts revealed a significant drop at T2 (0.44-point decrease, *p-adj* < 0.001). This decrease was maintained at T4 (0.46-point decrease, *p-adj* < 0.001). Similarly, the overall level of satisfaction did not differ among smokers and those who achieved abstinence (*b* = −0.14, *p* = 0.425), nor did the extent of decrease over time (interaction effect: *b* = 0.03, *p* = 0.507). These results suggest that satisfaction with the interaction sessions did not significantly influence smoking cessation outcomes.

### 3.4 Dropout analysis

The number of participants who completed each weekly session is detailed in Fig. 3. As shown, after some drop-out after the pre-quit session, retention rates remained relatively stable for the remainder of intervention. Overall, 56.9 % (58/102) of participants completed the chatbot intervention. We performed an exploratory logistic regression analysis to identify factors associated with dropout. However, none of the demographic variables or baseline smoking behavior appeared to be significant predictors of abstinence. Detailed results can be found in the supplementary file.

## 4 Discussion

### 4.1 Principal findings

In this study, we investigated the feasibility and potential usefulness of a chatbot-delivered, longitudinal smoking cessation intervention. Drawing on motivational interviewing and CBT techniques, we developed Roby to engage adult smokers in 5 chat sessions over multiple weeks. Our findings provide support for using a chatbot for long-term smoking cessation: by the end of the intervention, 18.6 % of participants (and 32.8 % of those who completed all sessions) achieved continuous abstinence, while 37.3 % (and 65.5 % of completers) achieved a 7-day PPA. Since existing meta-analyses on chatbot interventions did not provide comparable data over similar timeframes (Bendotti et al., 2023), we compared our results with other forms of cessation interventions in the Netherlands involving comparable samples and similar timelines, which allowed for tentative yet meaningful contextualization of our results. One web-based program incorporating value clarification techniques reported a 27 % 7-day PPA rate one month since the start of the intervention (Gültzow et al., 2022), while another study among Dutch smokers found a 7 % 7-day PPA rate in a waitlist group six weeks after starting (Smit et al., 2012). The comparison to the waitlist group serves as a benchmark to highlight potential benefits of chatbot interventions compared to minimal or delayed support, considering that chatbots can be particularly helpful for individuals on a waitlist because they provide immediate support, maintain user engagement during the waiting period, and help build motivation to quit through consistent interaction (Sharp et al., 2024; Richards et al., 2024). Although no definitive conclusions can be drawn without a randomized controlled trial, these comparisons suggest a promising outlook for chatbots as a tool for smoking cessation. Future studies could benefit from comparisons with more diverse geographical and cultural contexts to better evaluate the generalizability of such interventions. Additionally, our results showed significant improvements in self-efficacy and reductions in cravings – both key factors for successful quitting (Blevins et al., 2016). Notably, these improvements appeared to accumulate over the sessions. For instance, while there were no significant changes in self-efficacy between T1 and T2 (or T2 and T3), a significant increase was observed from T1 to T3. This suggests that a single session may not have an immediate effect, but the cumulative impact over time is meaningful. This aligns with previous research on multi-session motivational interviewing and chatbot interventions, which indicate that the effects of such interventions tend to build gradually over multiple sessions (Lindson-Hawley et al., 2015), highlighting the potential for using chatbot in long-term cessation support. Moreover, the content of the weekly sessions appears to play a role in the observed changes. The largest increase in self-efficacy occurred between T0 and T1, likely because Roby employed confidence-boosting strategies in the T0 session, such as discussing previous quit attempts and identifying personal strengths. This suggests that chatbot interventions should cover a broad range of topics and revisit them regularly for maximum impact.

To the best of our knowledge, this is the first study to explore the trajectory of user experience over several weeks, providing insights into how people's perceptions evolve over time. We found an overall decrease for engagement and user satisfaction, which may be attributed to a variety of factors, such as diminishing novelty of the chatbot (Wells et al., 2010) or reduced perceived relevance of the content as participants progressed in their quitting journeys. However, it is notable that this decline plateaued towards the later stages of the intervention. Specifically, the differences in engagement and satisfaction between T2 and T3, as well as T3 and T4, were not statistically significant. This finding suggests that while there was an initial drop-off, user experience stabilized as the intervention progressed. This stabilization complements the observed novelty effect and emphasizes the importance of actively monitoring and supporting engagement throughout the intervention. Moreover, while the overall decrease in engagement and satisfaction was statistically significant, it was not substantial (0.35 and 0.46 points on a 5-point scale, respectively). This indicates that the chatbot was generally well-received and able to maintain a reasonable level of user experience throughout the intervention. This implies that the chatbot has the potential to keep users engaged, even in the face of decreasing novelty. Similar patterns of engagement drop-off have been often observed in human-computer interactions and digital interventions (De Haas et al., 2022; Bickmore et al., 2010), calling for future research to look into strategies to maintain user engagement. For instance, an earlier content analysis of interactions with a smoking cessation chatbot identified cognitive involvement as a key driver of user engagement (He et al., 2024b). Future research could build on this by incorporating cognitive strategies, such as quizzes or games, to enhance and maintain user engagement throughout the intervention.

Moreover, we did not find significant changes in therapeutic alliance over the weeks, which remained moderate throughout the intervention. This moderate level suggests that while the chatbot provided consistent support, it may not have fully strengthened the user connection. Current chatbots might lack the capacity to form a meaningful human-chatbot bond, a key dimension of therapeutic alliance (Tong et al., 2022). Previous research has suggested that users often do not develop a sense of relationship with chatbots due to the lack of intimacy and empathy (Croes and Antheunis, 2021). Next to the bond dimension, research on digital therapeutic alliance highlighted the importance of supporting users completing tasks and achieving goals (Tong et al., 2022). Future chatbot design could consider incorporating strategies addressing this aspect, such as providing timely feedback and offering contingent rewards.

Despite the overall decreasing trend in user experience, this decline did not impact the outcomes of the intervention. The statistical analysis results showed that the general level of user experience and the extent of its decrease were similar between participants who achieved abstinence and those who did not. This suggests that while user experience may decrease over time, it does not necessarily affect the success of the intervention in terms of cessation. Previous research also noted that an app can lead to positive clinical outcomes without necessarily building an engaging and therapeutic alliance (Tong et al., 2022). Nevertheless, this does not imply that user experience should be overlooked. A more engaging user experience might lead to higher retention rates and a more positive perception of the chatbot, which could indirectly support better long-term outcomes (Limpanopparat et al., 2024).

### 4.2 Strengths and limitations

A major strength of this study is that it used a standalone, content-rich chatbot that incorporated MI and CBT in a fully automated manner. Additionally, while most previous chatbot interventions quantify engagement based on number or duration of the interactions, we tracked subjective user experience over time. Our longitudinal design provided a unique opportunity to study not only how often participants interacted with the chatbot, but also how their perceptions of the chatbot evolved throughout the intervention. This approach adds to existing literature by highlighting the dynamic nature of user engagement. Capturing this subjective dimension moves beyond traditional metrics and offers insights into how user experience affects long-term intervention outcomes.

This study has several limitations that should be considered. First, participants received incentives for participation, which could potentially influence their motivation and responses. While incentives help improve engagement and retention, they may also introduce biases in how participants report their experiences and outcomes. Second, the study did not include a control group as this was a proof-of-concept feasibility study. The absence of a control group limits our ability to make definitive conclusions about the intervention's effectiveness compared to other methods or to no intervention. Additionally, we used self-reported measures for abstinence, without biological verification. Moreover, our sample primarily included younger participants and light smokers who were motivated to quit within two weeks. This recruitment criterion may have attracted a more motivated group, who are more likely to adopt and follow the chatbot intervention. Given that we have established the feasibility and initial usefulness of the chatbot, future studies should validate these finding among broader and more diverse samples with full-scale randomized controlled trials (RCTs) including biological measures to provide more robust evidence.

An important aspect of our study is that the chatbot was pre-scripted, which presents both strengths and limitations. On the one hand, this ensured safety and reliability, crucial in the health domain. By using a pre-scripted approach, we minimized the risk of the chatbot providing untruthful, unreliable, or harmful information, thereby upholding the intervention's integrity. On the other hand, this controlled design may have limited the chatbot's flexibility and, consequently, impacted the user experience. Despite incorporating strategies to enhance adaptability, the balance between a rigid, controlled script and a more dynamic, personalized interaction remains challenging. Future developments should aim to find an optimal balance that maintains safety while enhancing user engagement and satisfaction.

### 4.3 Implications for future research and practice

Our findings provide suggestive evidence of the usefulness of a chatbot-delivered smoking cessation intervention, with notable improvements in self-efficacy and reductions in cravings. However, to confirm these effects and draw more definitive conclusions, robust RCTs are needed in future research. Moreover, while the user experience declined throughout the intervention, our findings indicate that this did not compromise the overall positive results. However, enhancing user experience remains an important goal, as it could potentially improve participant retention and foster a more positive interaction with the chatbot. Future studies should focus on strategies to maintain engagement over time, such as introducing interactive features or refreshing content to keep the experience dynamic and relevant.

Lastly, our study calls into question the traditional conceptualization of therapeutic alliance in the context of human-chatbot interactions. While emotional bonding with chatbots may be limited, future research could explore how task-oriented support, consistent feedback, and personalized interactions can redefine alliance in human-chatbot relationships, ensuring that digital agents can effectively support users in achieving their health goals (Herbener et al., 2024).

### 4.4 Conclusion

This study demonstrates the feasibility and potential usefulness of a chatbot intervention for smoking cessation. We observed an 18.6 % continuous abstinence rate, a 37.3 % 7-day PPA rate, significant improvements in self-efficacy, and reduced cravings. While user experience declined, likely due to novelty, this did not affect the intervention's outcomes. These findings highlight the promise of chatbot interventions for long-term cessation support and the need for continued evaluation through RCTs. Future efforts should focus on enhancing and maintaining user experience throughout the intervention to maximize its effectiveness.

## CRediT authorship contribution statement

L.H., E.K., M.A., and R.W. conceived the presented idea and designed the study. L.H. and E.B. developed the chatbot used in the experiment. L.H. carried out the study, collected and analyzed the data. L.H. drafted the main manuscript text and prepared the tables and figures. All authors contributed to the final version and approved the final manuscript.

## Ethical approval

The experiment was approved by the Research Ethics and Data Management Committee of the Tilburg School of Humanities and Digital Sciences (Identification code: REDC 2021.18d) and was conducted in compliance with the ethical and data management regulations of the school. Informed consent was obtained from all subjects.

## Funding

This work is supported by the Look Who's Talking Project (official project code: 406.D1.19.054), funded by the Dutch Research Council (NWO). The funder has no role in study design, data collection and analysis, decision to publish or preparation of the manuscript.

## Declaration of competing interest

The authors declare that they have no known competing financial interests or personal relationships that could have appeared to influence the work reported in this paper.

## Appendix A Supplementary data

Supplementary material Image 1

## Footnotes

1 While the original content was based on a manual-guided protocol for adolescents, necessary adaptations were made to ensure its applicability to adults. These adaptations included removing adolescent-specific elements (e.g., social pressure to smoke) and incorporating universally relevant topics such as coping strategies and emotional management.
