# Supplementary material for: Chatting your way to quitting: A longitudinal exploration of smokers' interaction with a cessation chatbot
Source: Internet Interv. 2025 Feb 4;39:100806. doi: 10.1016/j.invent.2025.100806 (PMC11847720; doi:10.1016/j.invent.2025.100806)
Supplement: Supplementary file 1 — Supplementary material [file mmc1.docx]

We conducted an exploratory logistic regression analysis to identify factors associated with achieving abstinence. However, none of the demographic variables or baseline smoking behavior appeared to be significant predictors of abstinence. See Table 1 below for detailed results.

Table 1. Logistic regression predicting continuous abstinence.

| Variable | *B* | Standard error *B* | *P* | Odds ratio |
| --- | --- | --- | --- | --- |
| Intercept | *-1.50* | 4.52 | *.740* | 0.22 |
| Age | 0.17 | 0.18 | .339 | 1.19 |
| Male | -0.38 | 0.68 | .579 | 0.49 |
| Education level_Medium | 0.51 | 0.92 | .578 | 1.67 |
| Years of smoking | -0.04 | 0.18 | .808 | 0.96 |
| Daily cigarette consumption | -0.03 | 0.14 | .826 | 0.97 |
| FTND ^a^ | 0.85 | 1.20 | .476 | 2.35 |
| Baseline motivation to quit | -0.10 | 0.17 | .562 | 0.91 |
| Daily smoker | 0.57 | 0.81 | .483 | 1.77 |
| Light smoker ^b^ | 0.50 | 1.86 | .787 | 1.65 |
| Self-efficacy_t4 | -0.54 | 0.52 | .300 | 0.58 |
| Craving_t4 | 0.14 | 0.40 | .734 | 1.15 |

^a^ Fagerström test for nicotine dependence. ^b^ Smokes 10 cigarettes or less per day.

We performed another exploratory logistic regression analysis to identify factors associated with dropout. However, as shown in Table 2 below, none of the demographic variables or baseline smoking behavior appeared to be significant predictors of abstinence.

Table 2. Logistic regression predicting dropout.

| Variable | *B* | Standard error *B* | *P* | Odds ratio |
| --- | --- | --- | --- | --- |
| Intercept | -1.19 | 2.66 | .656 | 0.31 |
| Age | 0.10 | 0.09 | .271 | 1.11 |
| Male | 0.11 | 0.49 | .830 | 1.11 |
| Education level_Low | 0.15 | 0.00 | .995 | <.001 |
| Education level_Medium | 0.37 | 0.60 | .535 | 1.45 |
| Years of smoking | -0.00 | 0.09 | .971 | 1.00 |
| Daily cigarette consumption | -0.07 | 0.11 | .513 | 0.93 |
| FTND ^a^ | -0.43 | 0.77 | .581 | 0.65 |
| Baseline motivation to quit | 0.11 | 0.11 | .335 | 1.12 |
| Daily smoker | 0.02 | 0.58 | .967 | 1.02 |
| Light smoker ^b^ | -2.54 | 1.35 | .061 | 0.08 |

^a^ Fagerström test for nicotine dependence. ^b^ Smokes 10 cigarettes or less per day.
